# Public health round-up

**DOI:** 10.2471/BLT.26.011126

**Published:** 2026-11-01

**United Nations *Decade on Healthy Ageing report***

A mid-term report on the United Nations (UN) Decade of Healthy Ageing (2021–2030) presented at the 81st UN General Assembly showed significant progress in improving the lives of older people, while warning that major gaps remain. More countries have introduced laws against age discrimination, strengthened long-term care policies and expanded age-friendly environments and integrated care services. However, financing remains a key challenge, with many countries lacking dedicated resources for long-term care and healthy ageing initiatives. With the global population aged 60 and over projected to double to 2.1 billion by 2050, the World Health Organization and partners are calling for increased investment, stronger data systems and accelerated action to ensure healthy ageing for all.

**Figure:**
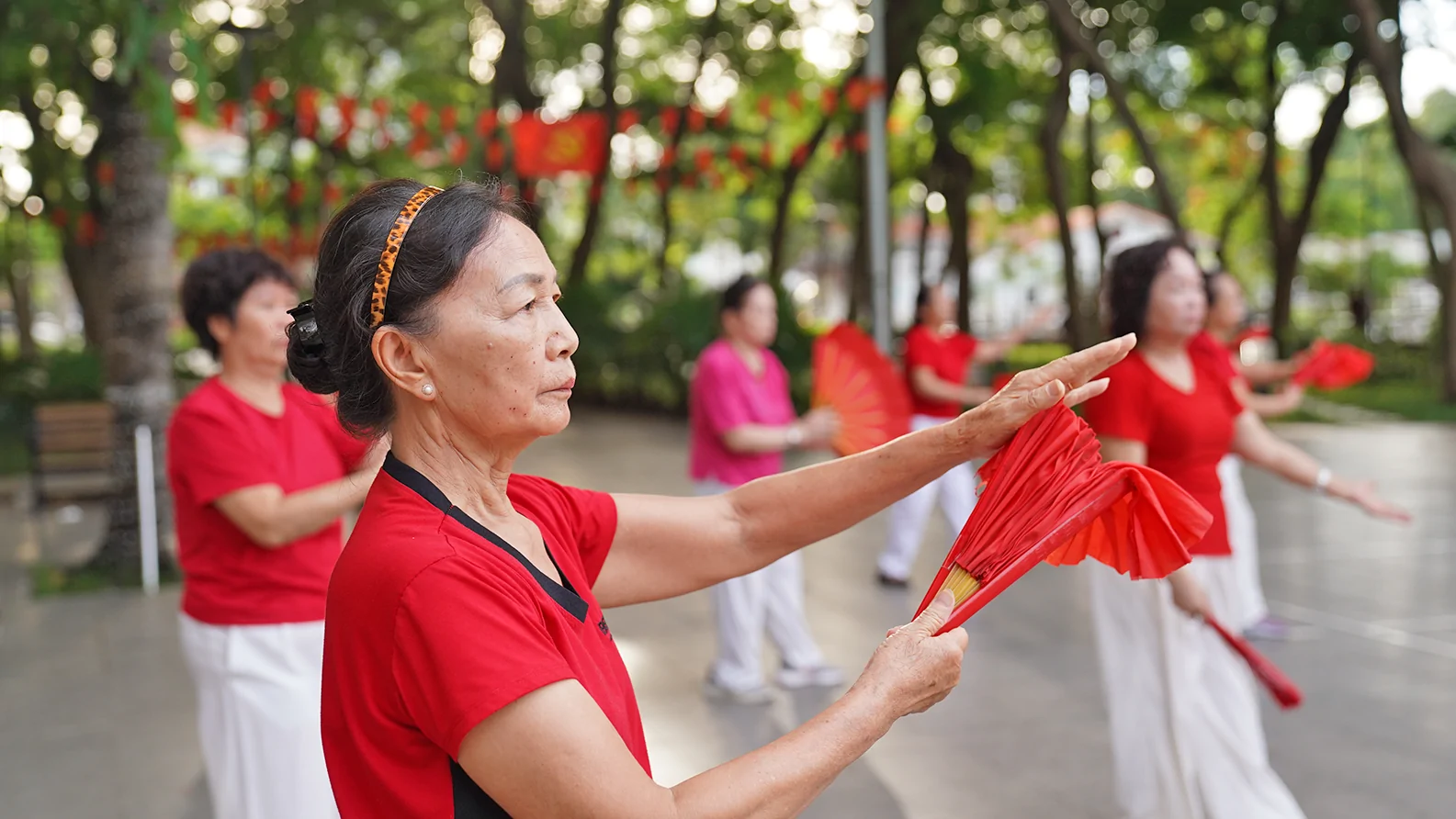
WHO/Ho Chi Cuong

## Political declaration for pandemic preparedness

The World Health Organization (WHO) has welcomed the adoption by the United Nations General Assembly (UNGA) of a new *Political declaration on pandemic prevention, preparedness and response*, reaffirming global commitment to protect future generations from the impacts of pandemics.

The declaration follows discussions at the second UNGA High-level Meeting on pandemic prevention, preparedness and response and builds on progress made since the COVID-19 pandemic, including the adoption of the WHO Pandemic Agreement. It calls for sustained investment to strengthen national, regional and global capacities to prevent, detect and respond to future health threats. Key priorities include enhancing surveillance systems, implementing the amended *International health regulations* (2005), supporting equitable access to vaccines and other health products, expanding regional manufacturing capacity and securing sustainable financing. Particular emphasis was placed on concluding negotiations on the Pathogen Access and Benefit Sharing (PABS) annex. 

“The world has come a long way since COVID-19. We urge countries to now fulfil the commitments they have made, especially to finalize negotiations on the PABS annex, so the WHO Pandemic Agreement can be opened for signature, begin the process of ratification and enter into force as international law,” said WHO Director-General Tedros Adhanom Ghebreyesus. 

https://bit.ly/47TuMCY

## Famine risk in Sudan

The Integrated Food Security Phase Classification (IPC) has warned of a growing risk of famine in parts of Sudan, particularly in Greater Darfur and Greater Kordofan, where ongoing conflict, mass displacement, severe drought and inadequate humanitarian funding are driving a rapidly worsening crisis. 

A drought affecting around half the country is further reducing food availability, increasing prices and undermining livelihoods. The alert highlights alarming levels of acute food insecurity and malnutrition while limited access to health care and clean water leaves vulnerable populations at heightened risk of disease and death. 

WHO continues to support health and nutrition services across Sudan, including treatment of children with severe acute malnutrition and response to outbreaks such as cholera and measles. Between January and August 2026, more than 26 000 children received treatment through nutrition stabilization centres supported by WHO. With only 23% of its humanitarian appeal funded, WHO is calling for increased funding, sustained humanitarian access and urgent action to prevent further deterioration of the situation.

https://bit.ly/4s41yuB

## Community-based mental health care

WHO has released a new guide to help countries transition from long-term institutional mental health care to person-centred, community-based services that respect people’s rights, dignity and choices. The guidance supports the process of deinstitutionalization, helping people who have spent extended periods in psychiatric hospitals return to their communities with access to housing, health care, social services, education and employment opportunities. *Supporting the transition to community-based mental health and social care* provides practical recommendations for reducing long-term hospital stays, strengthening rights-based care and supporting community reintegration. 

Despite growing efforts to expand community mental health services, progress remains uneven. According to WHO’s *Mental health atlas*, only 9% of reporting countries have fully transitioned to community-based care, while more than half remain in the early stages of reform. 

The guide also emphasizes the importance of involving people with lived experience in shaping mental health policies and services. “Shifting towards community-based mental health care must be done with people who know what it means to go through institutional care, not for them,” said Grace Gatera, independent lived experience consultant and advocate. “Their expertise, alongside that of others, is essential to building services that support choice, recovery and participation in community life.”

https://bit.ly/4ijPt0Y

## First childhood obesity guidelines

Responding to a sharp rise in obesity worldwide, WHO has released its first guidelines on the integrated management of obesity in children and adolescents. In 2024, an estimated 170 million people aged 5–19 years were living with obesity, with prevalence in this age group having quadrupled since 1990. 

The guidelines emphasize that obesity is a chronic disease requiring timely, evidence-based and person-centred care. WHO strongly recommends structured interventions combining healthy diets, physical activity and behaviour change, delivered individually or through comprehensive programmes. For younger children, the Organization does not recommend medicines, surgery or weight-loss devices, while these options may be considered for adolescents only under strict conditions.

The guidelines also highlight the importance of addressing mental health alongside obesity and call for broader policy measures to improve access to healthy diets and physical activity. “The foundation of obesity care for children and adolescents is not medicine or surgery, but access to comprehensive support that enables healthy eating, physical activity and sustainable behaviour change,” said Luz María De Regil, director of WHO’s Department of nutrition and food safety.

https://bit.ly/4hM6SPF

## Together for science and health

WHO has launched a global call for organizations and institutions to join Together for science and health, a new movement aimed at strengthening the relationship between science, health and society and building trust in evidence-informed decision-making. The initiative recognizes the transformative role of science in improving health, from advances in vaccination and antimicrobial treatments to progress in HIV care, cancer prevention and child survival. It seeks to bring together researchers, health professionals, policy-makers, communities, funders and citizens to ensure that scientific knowledge is translated into better health outcomes for all. 

Built around three pillars, science and society, science and policy, and science communication, the movement will promote collaboration across sectors, support the use of evidence in health decision-making and encourage meaningful engagement with communities. It also aims to improve public understanding of science and strengthen responses to misinformation and disinformation. 

https://bit.ly/4rZGXaB

## Global health estimates

Global life expectancy has largely recovered to pre-pandemic levels, reaching 73.3 years in 2023, according to WHO’s latest *Global health estimates*. However, healthy life expectancy has recovered more slowly, highlighting the need not only to extend life, but also to improve health in later years. 

The new estimates, covering trends from 2000 to 2023, show a continuing shift in the global burden of disease towards noncommunicable diseases, which accounted for 74% of all deaths in 2023, up from 58% in 2000. Cardiovascular diseases remain the leading cause of death worldwide, while diabetes, dementia, mental health conditions and drug use disorders are becoming increasingly important contributors to ill health and mortality. For the first time, communicable diseases accounted for less than half of all deaths in low-income countries, reflecting a major epidemiological transition. 

“Living longer is one of the great achievements of public health,” said Alain Labrique, director of WHO’s Department of data, digital health, analytics and AI. “The next challenge is to ensure that those additional years are lived in good health, while health systems are equipped to respond to the changing needs of populations.”

https://bit.ly/4i1FDzC

## Tobacco tax reform results

Recent tobacco tax reforms in low- and middle-income countries are demonstrating how higher excise taxes can reduce smoking while generating revenue for health and development. A new analysis of 15 countries found that all improved cigarette taxation between 2009 and 2025, with the strongest results achieved through substantial tax increases, simpler tax systems and measures to prevent tobacco products from becoming more affordable over time. 

The Philippines recorded real cigarette price increases of more than 600% and noted that tobacco-tax reforms helped finance the expansion of universal health coverage. Bangladesh, Brazil, India, Mexico and Pakistan also reported notable declines in tobacco use. 

The findings support WHO’s 3 by 35 Initiative, which encourages countries to increase the real prices of tobacco, alcohol and sugary drinks by 2035 to reduce noncommunicable diseases and create sustainable domestic resources for health and development

https://bit.ly/3W8WJ7f

**Cover photo**

Maru recycles used lead-acid batteries at his workshop in Lideta, Addis Ababa, Ethiopia. Recycling used lead-acid batteries is a primary source of global lead contamination and human exposure.

**Figure:**
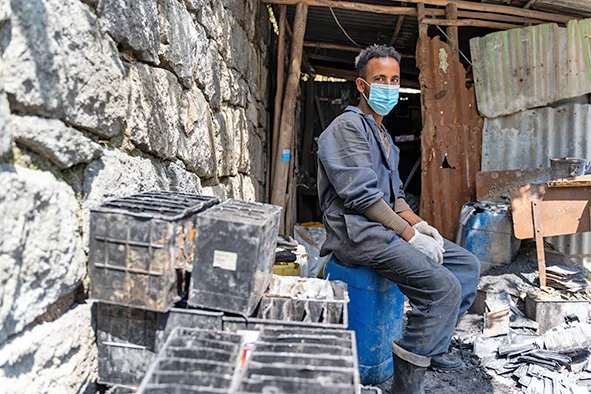
WHO/ Addis Aemero

**Looking ahead**

10 November. Together for Science and Health, launch event. https://bit.ly/3Wove9O

14 November. World Diabetes Day. https://bit.ly/4yJjQUj

17 November. World Cervical Cancer Elimination Day. https://bit.ly/4hXGIbB

